# Mapping of the evidence from systematic reviews of the Cochrane Collaboration for decision-making within physiotherapy

**DOI:** 10.1590/S1516-31802013000100007

**Published:** 2013-02-01

**Authors:** Ane Helena Valle Versiani, Ana Cabrera Martimbianco, Maria Stella Peccin

**Affiliations:** I PT. Physiotherapist and Master’s Student in the Postgraduate Program on Internal Medicine and Therapeutics, Universidade Federal de São Paulo (Unifesp), São Paulo, Brazil.; II PT. Physiotherapist and Preceptor of the Hospital Sector of the Specialization Course on Outpatient and Hospital Motor Physiotherapy applied to Orthopedics and Traumatology, Universidade Federal de São Paulo (Unifesp), São Paulo, Brazil.; III PT, PhD. Professor, Department of Human Movement Sciences, Universidade Federal de São Paulo (Unifesp), Santos, São Paulo, Brazil.

**Keywords:** Evidence-based practice, Physical therapy modalities, Randomized controlled trials as topic, Review [publication type], Intervention, Prática clínica baseada em evidências, Modalidades de fisioterapia, Ensaios clínicos controlados aleatórios como assunto, Revisão, Estudos de intervenção

## Abstract

**CONTEXT AND OBJECTIVE:**

Evidence-based clinical practice emerged with the aim of guiding clinical issues in order to reduce the degree of uncertainty in decision-making. The Cochrane Collaboration has been developing systematic reviews on randomized controlled trials as high-quality intervention study subjects. Today, physiotherapy methods are widely required in treatments within many fields of healthcare. Therefore, it is extremely important to map out the situation regarding scientific evidence within physiotherapy. The aim of this study was to identify systematic reviews on physiotherapeutic interventions and investigate the scientific evidence and recommendations regarding whether further studies would be needed.

**TYPE OF STUDY AND SETTING:**

Cross-sectional study conducted within the postgraduate program on Internal Medicine and Therapeutics and at the Brazilian Cochrane Center.

**METHODS:**

Systematic reviews presenting physiotherapeutic interventions as the main investigation, in the Cochrane Reviews Group, edition 2/2009, were identified and classified.

**RESULTS:**

Out of the 3,826 reviews, 207 (5.41%) that fulfilled the inclusion criteria were selected. Only 0.5% of the reviews concluded that the intervention presented a positive effect and that further studies were not recommended; 45.9% found that there seemed to be a positive effect but recommended further research; and 46.9% found that the evidence was insufficient for clinical practice and suggested that further research should be conducted.

**CONCLUSION:**

Only one systematic review (“Pulmonary rehabilitation for chronic obstructive pulmonary disease”) indicated that the intervention tested could be used with certainty that it would be effective. Most of the systematic reviews recommended further studies with greater rigor of methodological quality.

## RESUMO

**CONTEXTO E OBJETIVO:**

A prática clínica baseada em evidências surgiu com o intuito de guiar as questões clínicas para reduzir o grau de incerteza na tomada de decisão. A Colaboração Cochrane vem desenvolvendo revisões sistemáticas de ensaios clínicos controlados aleatórios como assunto de estudos de intervenção de alta qualidade. Atualmente, as modalidades de fisioterapia têm sido amplamente requisitadas nos tratamentos em diversas áreas da saúde. Portanto, a realização do mapeamento sobre a situação das evidências científicas da fisioterapia é de extrema importância. O objetivo do estudo foi identificar as revisões sistemáticas e verificar a evidência científica das intervenções fisioterapêuticas e a recomendação ou não de mais estudos.

**TIPO DE ESTUDO E LOCAL:**

Estudo transversal, realizado no programa de pós-graduação em Medicina Interna e Terapêutica e no Centro Cochrane do Brasil.

**MÉTODOS:**

Foram identificadas e classificadas as revisões sistemáticas que apresentavam intervenções fisioterapêuticas como investigação principal, nos grupos da “Cochrane Reviews Group”, edição 2/2009.

**RESULTADOS:**

Das 3.826 revisões, foram selecionadas 207 (5,41%) que preencheram os critérios de inclusão. Apenas 0,5% das revisões concluíram que a intervenção apresenta efeito positivo e não são recomendados mais estudos; 45,9% mostraram que a intervenção parece ter efeito positivo, e mais pesquisa é recomendada; em 46,9% das revisões, a evidência era insuficiente para prática clínica e foi sugerida mais pesquisa.

**CONCLUSÃO:**

Apenas uma revisão sistemática, *“Pulmonary rehabilitation for chronic obstructive pulmonary disease”*, indica o uso da intervenção testada com certeza de sua efetividade. A maioria das revisões sistemáticas recomendam estudos futuros com mais rigor na qualidade metodológica.

## INTRODUCTION

With improving means of communication and the advent of the internet, information has become more accessible to the population and the volume of scientific production has been increasing. New approaches and techniques for healthcare management have emerged, thus bringing in doubts regarding the best options in choosing treatments.^1^ This has forced healthcare professionals to seek up-to-date information for evaluating, diagnosing, preventing and treating clinical conditions.

This information can be obtained through a variety of media, such as books, congress proceedings, periodicals, printed or electronic journals, websites, CD-ROMs and DVDs, among others. However, it would be impossible to read all the articles, in view of the large numbers published every year. Moreover, among these, there will be articles of higher or lower quality, greater or lower trustworthiness and greater or lesser pertinence.^2^ Thus, it is not enough to seek the sources that are available: there is a need to know how to select from this information, so as to obtain the best and most reliable scientific evidence that provides answers for a given clinical question, so that healthcare decisions can be made.

Evidence-Based Medicine or Evidence-Based Practice arose in the 1990s, with the aim of guiding clinical questions such that the degree of uncertainty in making clinical decisions would be reduced.^3^ It is defined as clear, informed and rigorous use of the best and most up-to-date research evidence in making clinical decisions on patient care.^4^ It is practiced based on combining clinical experience with the best evidence available and patients’ preferences,^5^^,^^6^^,^^7^ which allows clinicians to keep themselves up-to-date and become more critical, thereby improving the degree of confidence in their judgments.^5^

Nonetheless, the best evidence is not immutable. In other words, it may undergo changes through production of new studies of better quality.^8^^,^^9^

It is now recognized that randomized clinical trials are the most appropriate type of study for proving whether a given intervention is effective. However, the results are often insufficient to provide a clear answer for the clinical question. When there is a possibility of grouping these studies together, the conclusions may become clearer and more objective, with a tendency towards improvement of their reliability and accuracy.^10^^,^^11^

Systematic reviews are efficient and reproducible scientific investigations on original studies, using preplanned methodology.^12^ They may help professionals to keep abreast of the scientific literature through summarizing large quantities of information covering a specific clinical question, since these reviews provide a synthesis of the results from several primary investigations, using strategies that limit the degree of bias.

The Cochrane Collaboration has taken on a commitment to produce syntheses from randomized clinical trials on healthcare interventions.^13^ Its main work consists of compiling systematic reviews, which are conducted by Collaborative Review Groups and inserted in the Cochrane Library.^7^^,^^14^^,^^15^

The conclusions from a review depend both on the quality of the randomized clinical trials and on the quality of the planning process for the review.^16^ Cochrane reviews include methods for minimizing bias, such as a classification system for judging the methodological quality and absence of language restrictions.^17^^,^^18^ For such reasons, they are considered to be the gold standard for evidence-based healthcare.

Over recent years, physiotherapy^19^^,^^20^^,^^21^^,^^22^^,^^23^ has been widely required in a variety of fields of healthcare. This has made it clear that there is a need for scientific proof regarding the effectiveness of the different types of treatment used in the rehabilitation process. Therefore, it is extremely important to map out the situation relating to scientific evidence within physiotherapy.

## OBJECTIVE

The aim of the present study was to identify the complete systematic reviews produced by the Cochrane Collaboration that involve physiotherapeutic treatments and to investigate whether the scientific evidence was sufficiently strong for no further studies to be needed, or whether there was a recommendation for further studies in order to achieve certainty that the intervention was effective.

## METHODS

In this study, the 51 groups of the Cochrane Collaboration (“Cochrane Review Groups”) in the Cochrane Library, edition 2, 2009, were analyzed by means of the CD-ROM of the Wiley InterScience database, and complete systematic reviews that presented any physiotherapeutic interventions as the main investigation were selected.

The reviews were identified and selected, group by group, by two independent investigators (AHVV and ACM). In situations of non-concordance, the discrepancy was resolved by a third investigator (MSP). The reviews were identified and gathered firstly according to their titles. In any cases of doubt regarding the content, the abstract or the entire text was read for clarification. The systematic reviews that were considered to be of interest were those that presented physiotherapeutic interventions within the fields of respiratory, musculoskeletal and neuromuscular physiotherapy, among others.

The reviews selected were entered into an Excel spreadsheet and were organized according to the Cochrane Collaboration groups. The spreadsheet was then organized according to the following items: group, title, objective, intervention, outcome, author’s conclusion, implications for practice, implications for research and classification.

Two independent investigators classified each review as presented below, using the information in the spreadsheet. The classification was based especially on the following items: author’s conclusion, implications for clinical practice and implications for research.

### Descriptions of the classifications and codes

1. Intervention has a positive effect and the author does not recommend further studies.
These were systematic reviews with conclusions presenting sufficient scientific proof to attest that the use of a given intervention was effective, such that its use could be recommended without any need for new studies.
2. Intervention seems to have a positive effect and the author recommends more research.
These were reviews in which the conclusion presented scientific proof supporting the use of the intervention investigated, thus suggesting that it was effective, but the authors were unsure of its benefit, in comparison with the control group, and recommended further studies to obtain better evidence.
3. Intervention has a negative effect and the author does not recommend more research.
These were reviews that presented sufficient scientific proof to contraindicate the use of the intervention in question, such that the authors were sure that the intervention was harmful and/or had adverse effects, in comparison with the control group, and that its use was not recommendable, without any need for further studies.
4. Intervention seems to have a negative effect and the author suggests further studies.
These were reviews that presented scientific proof that opposed the use of the intervention tested, although the authors were unsure of the harm caused, in comparison with the control group, and therefore suggested that further studies should be conducted to achieve greater certainty regarding the effect from the intervention.
5. Insufficient evidence for clinical practice and the author suggests more research.
These were reviews that did not present scientific proof that an intervention was more beneficial or more harmful, in comparison with the control group. In the conclusions, the authors used phrases like: “there was insufficient evidence to support or disprove”, “there was insufficient proof”, “no studies fulfilling the inclusion criteria were found” or “there was no evidence for coming to any conclusion”, among others. The authors thus encourage further research to cover the specific question.
6. Insufficient evidence to clinical practice and the author does not suggest more research.
These were reviews that did not present scientific proof that the intervention was beneficial or harmful, in comparison with the control group, but the authors did not suggest that further research should be conducted, thereby discouraging the production of new studies on the same question.

The concordance between the observers in performing the selection and classification was calculated using the kappa method.

## RESULTS

Among the 3,826 complete systematic reviews distributed across the 51 review groups of the Cochrane Collaboration, edition 2, 2009, only 207 reviews (5.41%) presented physiotherapeutic interventions as the main investigation, and these belonged to just 35 of the review groups. Of these 207 reviews, 11 were excluded because they were out of date and had been removed from edition 2, 2009, the publication that was the focus of our study.

In the selection process, 12 reviews of the 3.826, did not obtain agreement between AHVV and ACM and 4 were excluded by MSP. Among the 207 reviews evaluated and selected, 40 revisions (19.32%) did not achieve the same classification given by AHVV and ACM and were evaluated and classified by MSP.

After the investigators (AHVV and ACM) had selected and classified the reviews, the inter-observer concordance level was calculated. Kappa values of 0.9691 (Table 1) and 0.6311 (Table 2), respectively, for selection and classification were obtained.

**Table 1.** Distribution of the data for the kappa concordance test between the investigators AHVV and ACM regarding their selections of reviews. Kappa statistic = 0.9691

		ACM’s choices		
		Yes	No	Total
AHVV’s choices	Yes	199	7	206
	No	5	3615	3,620
	Total	204	3,622	3,826

ACM = Ana Cabrera Martimbianco; AHVV = Ane Helena Valle Versiani.

**Table 2.** Distribution of the data for the kappa concordance test between the investigators AHVV and ACM regarding their classifications of reviews. Kappa statistic = 0.6311

	Classification by ACM				
		Classification 2	Classification 4	Classification 5	Total
Classification by AHVV	Classification 1	1	0	0	1
	Classification 2	83	0	7	90
	Classification 3	0	0	0	0
	Classification 4	3	5	3	11
	Classification 5	22	2	68	92
	Classification 6	0	1	1	2
	Total	109	8	79	196

ACM = Ana Cabrera Martimbianco; AHVV = Ane Helena Valle Versiani.

Only one review, with the title “Pulmonary rehabilitation for chronic obstructive pulmonary disease”,^24^ belonging to the Cochrane Airways Group, received the classification 1. In the authors’ opinion, there were strong arguments confirming that respiratory rehabilitation was beneficial with regard to improvement of the quality of life observed at the start of the program, in comparison with the control group, and that there was no need for additional studies comparing respiratory rehabilitation and conventional community care among patients with chronic obstructive pulmonary disease. This review represented 0.5% of all the reviews classified.

The proportion of the reviews that presented interventions that seemed to show positive evidence, in comparison with the control group, but for which the authors recommended further research, was 45.9% (90 complete systematic reviews).

None of the systematic reviews was placed in classification 3 (Intervention has a negative effect and the author does not recommend more research).

The proportion of the reviews in which the intervention seemed to have a negative effect in comparison with the control group but the authors recommended further research was 5.6% (11 complete systematic reviews).

The outcome showing the greatest proportion (46.9%) was the one in which there was insufficient evidence to suggest any benefit or harmful effect in comparison with the control and the authors recommended further research on the intervention in question, which accounted for 92 complete systematic reviews.

Only two systematic reviews were placed in classification 6 (Insufficient evidence for clinical practice and the author does not suggest more research), which corresponded to 1.0% of all of the reviews.

**Graph 1.**
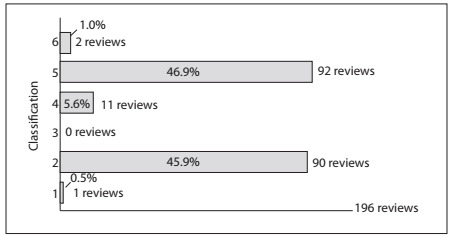
Absolute and relative percentage frequencies of Cochrane Collaboration systematic reviews on physiotherapy interventions in edition 2, 2009, according to classification.

Graph 1 shows the distribution of the absolute and relative frequencies of the complete systematic reviews that were selected and classified.

Table 3 shows the distribution of the absolute frequencies of the complete systematic reviews that were selected and classified by the groups of the Cochrane Collaboration, in edition 2, 2009.

**Table 3.** Distribution of absolute frequencies of complete systematic reviews selected and classified, according to the Cochrane Collaboration group, in edition 2, 2009

		CLASSIFICATION						Total
	GROUP	1	2	3	4	5	6	
1	Cochrane Acute Respiratory Infections Group	0	1	0	1	1	0	3
2	Cochrane Airways Group	1	4	0	0	7	0	12
3	Cochrane Anaesthesia Group	0	1	0	0	1	0	2
4	Cochrane Back Group	0	12	0	3	5	0	20
5	Cochrane Bone, Joint and Muscle Trauma Group	0	10	0	1	9	1	21
6	Cochrane Breast Cancer Group	0	1	0	0	1	0	2
7	Cochrane Cystic Fibrosis and Genetic Disorders Group	0	2	0	1	2	0	5
8	Cochrane Dementia and Cognitive Improvement Group	0	2	0	0	3	0	5
9	Cochrane Depression, Anxiety and Neurosis Group	0	2	0	0	3	0	5
10	Cochrane Developmental, Psychosocial and Learning Problems Group	0	3	0	0	1	0	4
11	Cochrane Ear, Nose and Throat Disorders Group	0	1	0	0	0	0	1
12	Cochrane Epilepsy Group	0	0	0	0	1	0	1
13	Cochrane Eyes and Vision Group	0	0	0	0	1	0	1
14	Cochrane Heart Group	0	4	0	0	1	0	5
15	Cochrane HIV/AIDS Group	0	2	0	0	0	0	2
16	Cochrane Hypertension Group	0	0	0	0	1	0	1
17	Cochrane Incontinence Group	0	2	0	0	5	0	7
18	Cochrane Infectious Diseases Group	0	0	0	0	1	0	1
19	Cochrane Inflammatory Bowel Disease and Functional Bowel Disorders Group	0	0	0	0	1	0	1
20	Cochrane Injuries Group	0	0	0	0	2	0	2
21	Cochrane Lung Cancer Group	0	1	0	0	0	0	1
22	Cochrane Menstrual Disorders and Subfertility Group	0	2	0	0	3	0	5
23	Cochrane Metabolic and Endocrine Disorders Group	0	2	0	0	1	0	3
24	Cochrane Movement Disorders Group	0	1	0	0	3	0	4
25	Cochrane Multiple Sclerosis Group	0	2	0	0	1	0	3
26	Cochrane Musculoskeletal Group	0	19	0	4	3	0	26
27	Cochrane Neonatal Group	0	0	0	1	4	1	6
28	Cochrane Neuromuscular Disease Group	0	2	0	0	9	0	11
29	Cochrane Pain, Palliative and Supportive Care Group	0	6	0	0	3	0	9
30	Cochrane Peripheral Vascular Diseases Group	0	2	0	0	0	0	2
31	Cochrane Pregnancy and Childbirth Group	0	3	0	0	6	0	9
32	Cochrane Schizophrenia Group	0	0	0	0	1	0	1
33	Cochrane Stroke Group	0	1	0	0	9	0	10
34	Cochrane Tobacco Addiction Group	0	2	0	0	0	0	2
35	Cochrane Wounds Group	0	0	0	0	3	0	3
		TOTAL						196

Classification 1 = Intervention has a positive effect and the author does not recommend further studies; Classification 2 = Intervention seems to have a positive effect and the author recommends more research; Classification 3 = Intervention has a negative effect and the author does not recommend more research; Classification 4 = Intervention seems to have a negative effect and the author suggests further studies; Classification 5 = Insufficient evidence for clinical practice and the author suggests further research; Classification 6 = Insufficient evidence for clinical practice and the author does not suggest more research.

## DISCUSSION

This study mapped out the complete systematic reviews that presented any physiotherapy intervention as the main arm of the investigation, distributed across the 51 groups of the Cochrane Collaboration in edition 2, 2009. It quantified the frequency of uncertainties and occurrences of lack of evidence to support or even to oppose the use of given physiotherapeutic interventions in different clinical situations.

However, out of all the reviews evaluated, only one of them, with the title “Pulmonary rehabilitation for chronic obstructive pulmonary disease”, belonging to the Cochrane Airways Group received classification 1 (Intervention has a positive effect and the author does not recommend further studies). Thus, this review confirmed that the intervention evaluated could be used safely and effectively for the specific clinic question examined. On the other hand, the reviews presenting classification 2 (Intervention seems to have a positive effect and the author recommends more research) accounted for 45.9% of the complete systematic reviews.

Furthermore, 46.9% of the reviews were placed in classification 5 (Insufficient evidence for clinical practice and the author suggests further research). These results are comparable with those from another study^25^ that evaluated Cochrane reviews in a general manner, thus showing that in terms of evidence, physiotherapeutic treatment interventions do not present results that are very different from interventions such as drug therapy, surgery and other interventions. This study also demonstrated that a high proportion of studies lack evidence to support or oppose their treatments.^25^

Thus, the majority of systematic reviews on physiotherapeutic treatment interventions lack sufficient evidence to provide answers for specific clinical questions.

Perhaps if we had considered any type of study, our result might have been different, but in questioning the effectiveness of a treatment or preventive intervention, we started from the presupposition that the most appropriate manner of addressing this would be to exclude non-experimental studies, since the results from such studies could furnish false positive conclusions regarding their efficacy and effectiveness. In this light, systematic reviews on randomized controlled clinical trials are recommendable, since the latter is the most appropriate type of study for answering clinical questions regarding treatment, given that the methodology of randomized clinical trials is more rigorous, with lower likelihood of bias, and is thus considered to be the gold standard.

A large proportion of systematic reviews do not have sufficient evidence to absolutely guarantee certain interventions. One of the problems detected by review authors concerns the methodological flaws of the primary studies or the lack of such studies. Most review authors suggest that new studies should be conducted and that the norms or recommendations for communication and publication of reports suggested by Consort (Consolidated Standards of Reporting Trials) should be used.^26^

The strategy used to identify relevant studies that will form part of a review, the methodology for evaluating these studies and the methods for grouping the results may affect the conclusions from systematic reviews. For this reason, for our research, we used the systematic review standards of the Cochrane Collaboration because of their extremely high level and methodological rigor. Cochrane reviews combine the best scientific studies in the world and are recognized as the gold standard for evidence-based healthcare. However, they depend on their primary studies, which need to be of better quality so that their evidence is sufficiently strong for there to be a consensus.

Our findings also demonstrated that only 1.0% of the reviews were placed in classification 4 (Intervention seems to have a negative effect and the author suggests further studies), and this low percentage may be related to publication bias. Many investigations that obtained negative results are not submitted for publication.^27^

One important point in this study was that it identified the complete systematic reviews that presented physiotherapeutic interventions as the main arm of the investigation, among the 3,826 complete reviews produced by the 51 groups of the Cochrane Collaboration. This selection was performed successfully, since the kappa statistical test for inter-observer concordance demonstrated almost perfect concordance, through obtaining the value of 0.9691. Any reviews for which concordance was not obtained were checked by a third investigator, who defined whether these reviews should be included or excluded. This was a means for retrieving the selection in a more trustworthy manner.

Out of the 3,826 complete systematic reviews found, only 207 reviews presented physiotherapeutic interventions as the main investigation, corresponding to 5.41% of the complete Cochrane Collaboration reviews in edition 2, 2009. This percentage may seem to be minute, but it is important to emphasize that physiotherapy is a relatively new field in evidence-based research.^21^ Moreover, although many scientific articles on physiotherapy are being produced, only a few clinical trials have yet been produced.^21^^,^^28^^,^^29^

One critical point in the present study was the difficulty that the investigators had in classifying reviews into certain categories, since many reviews were broad-based and took into consideration several treatment interventions in the same review, thus creating difficulty in reaching a final conclusion from the review, in relation to the original question. However, for classification purposes, the present investigators took into consideration the conclusion, the implication for research and the implication for clinical practice stated by the authors of each review.

The final result from this classification was assured by independent choices made by two investigators (AHVV and ACM), and it was observed that there was substantial concordance between them (kappa = 0.6311). Most of the reviews for which concordance between the evaluators was not obtained were placed either in classification 2 (Intervention seems to have a positive effect and the author recommends more research) or in classification 5 (Insufficient evidence for clinical practice and the author suggests further research). In interpreting the reviews between lack of evidence and the semblance of a positive effect, the evaluators often had doubts, considering that whereas some authors’ conclusions stated that there was insufficient evidence (variously because of a small sample size or poor, weak or inferior methodological quality), these authors sometimes stated in relation to implications for clinical practice that there was a small positive effect from the intervention.

Another problem observed was that many reviews considered several treatment interventions within a single review, such as exercise, surgery or medications, among others within the inclusion criteria. Attention needed to be given to this matter, since only the physiotherapeutic treatment interventions were of interest here.

In the light of the final results from the present study, we can say that physiotherapists and other healthcare professionals have the duty and responsibility to provide information for their patients in relation to the physiotherapeutic treatments for which scientific proof already exists, the treatments for which doubts regarding their effectiveness still exist and the treatments for which no scientific evidence exists yet.

Physiotherapists should also be prepared to critically evaluate the best evidence available, evaluate the results from their actions and observe with caution the therapeutic techniques and methods that present uncertainties. To assure and ensure good-quality healthcare, they should take into consideration three basic factors: their clinical experience, the best evidence currently available and the patient’s wishes and preferences.^30^

Thus, the present study affirms that there is a great need to conduct new primary studies of good quality, taking into consideration the methodological standardization and rigor of different physiotherapeutic interventions in various clinical situations and healthcare fields, in order to furnish conclusions of greater precision.

## CONCLUSIONS

Based on our results, there is only one systematic review (“Pulmonary rehabilitation for chronic obstructive pulmonary disease”,^24^ belonging to the Cochrane Airways Group) that really indicates the suitability of the intervention tested in comparison with the control group, with certainty that the intervention will be effective.

A significant proportion (46.9%) of the systematic reviews present doubts regarding the benefit or harm of their interventions, in comparison with the control group, thus confirming that there is insufficient scientific evidence to support or oppose such interventions in certain clinical situations.

An important proportion (45.9%) of the systematic reviews found that their interventions were beneficial in certain clinical situations, but recommended further studies with greater standardization and rigor of methodological quality.

In some specific fields of healthcare, there is still a lack of systematic reviews relating to physiotherapeutic treatments, which opens up a precedent for physiotherapeutic action needed in these areas and also for more studies to be conducted.
